# Intrathoracic liver herniation after pericardial fenestration – a case report

**DOI:** 10.1186/s13019-023-02282-6

**Published:** 2023-04-28

**Authors:** Peter Dubovan, Miroslav Tomáš, Jana Pavlendová, Ramadan Aziri, Marek Makovník, Jozef Dolník, Daniel Pinďák

**Affiliations:** 1 Department of Surgical Oncology, National Cancer Institute in Bratislava, Slovak Medical University in Bratislava, Bratislava, 833 10 Slovakia; 2 Department of Radiology, National Cancer Institute in Bratislava, Bratislava, 833 10 Slovakia

**Keywords:** Postoperative complication, Pericardial window, Liver, Hernia, Case report

## Abstract

**Background:**

Pericardial effusions with its potential life threatening progression towards cardiac tamponade have to be often managed with surgical intervention. In our case study we describe a complication after a common surgical procedure which has only scarce literature mentions.

**Case presentation:**

We present a case of a 22-year-old male patient who underwent subxiphoidal pericardial fenestration, due to symptomatic pericardial effusion with the Chamberlain procedure and biopsy of enlarged mediastinal lymph nodes. The histology report confirmed classical Hodgkin lymphoma and subsequently the patient underwent oncological treatment. Later on he was admitted to the hospital with dyspnoea and chest pain. The initial examinations stated a suspicion for intrathoracic tumour arising from the pericardium or liver. Further investigation revealed symptomatic intrathoracic liver herniation for which the patient underwent laparoscopic surgery with the mobilisation of liver and placement of a perforated Parietene^™^ composite mesh.

**Conclusion:**

The purpose of this case report is to describe a rare complication after pericardial fenestration with its potential clinical implications.

## Introduction

Pericardial effusion may arise due to a variety of reasons, which can be mostly divided into inflammatory and non-inflammatory. In some patients this can be one of the first signs of underlying malignant disease [1]. The subsequent management ranging from medical intervention to surgical procedure depends on the severity of symptoms and potential sings of cardiac tamponade. Surgical interventions bear potential risk of both early and late surgical complications, with some being more prevalent than others. Incisional hernias are common complications of abdominal surgery with the prevalence ranging from 2–20% [2]. However hernia complication associated with cardiothoracic surgery are less well reported [3]. The estimated incidence of subxiphoid incisional hernia after median sternotomy is 1–4%, while diaphragmatic hernias are mostly regarded as congenital or arising due to a traumatic event [3]. There is a scarcity of papers describing diaphragmatic herniation as a complication of cardiac surgery. This report describes such a clinical scenario with the intrathoracic liver protrusion. This case report has been reported in line with the SCARE (Surgical CAse REport) criteria [4].

## Case presentation

**Fig. 1:**
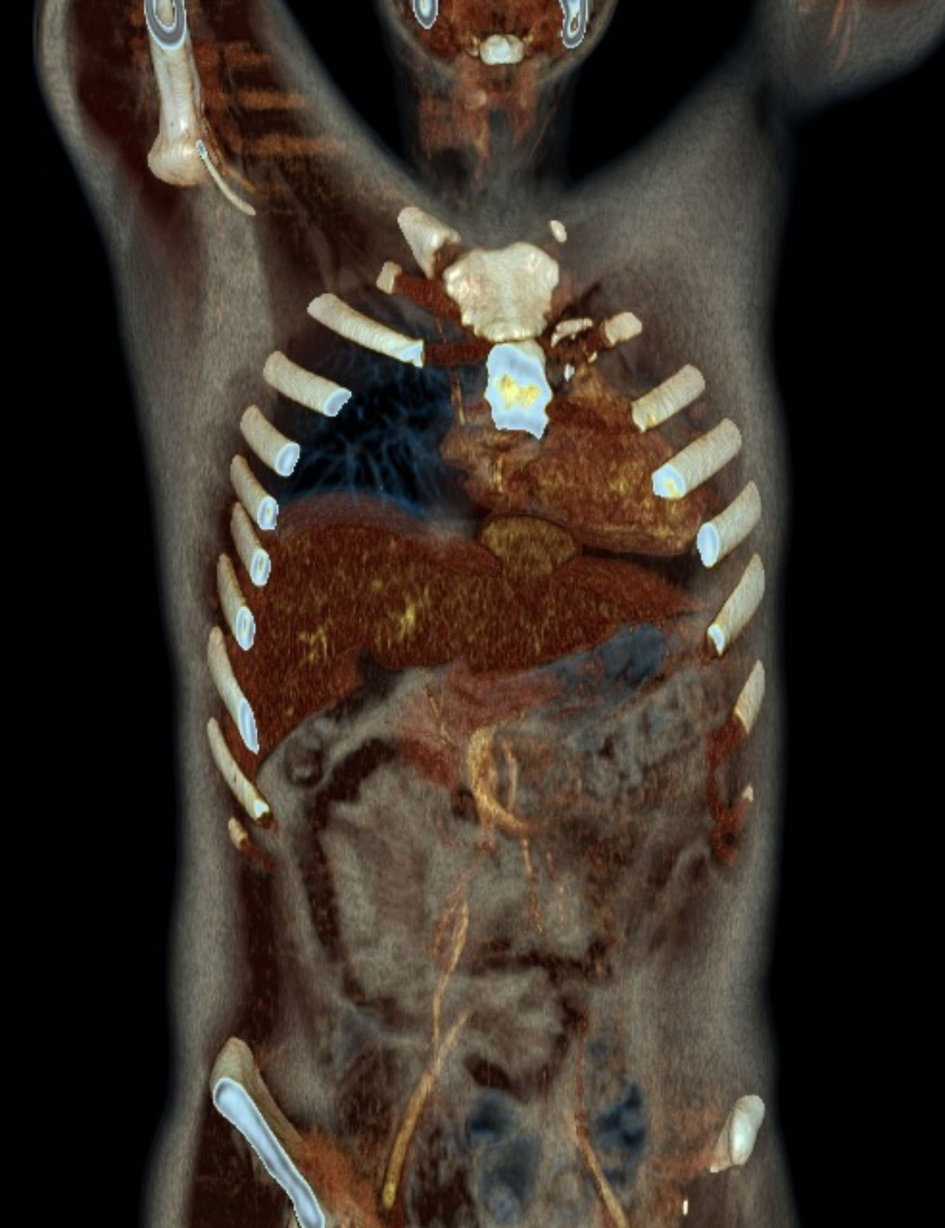
3D reconstruction of PET/CT scans with the main findings of liver herniation into the thoracic cavity

**Fig. 2:**
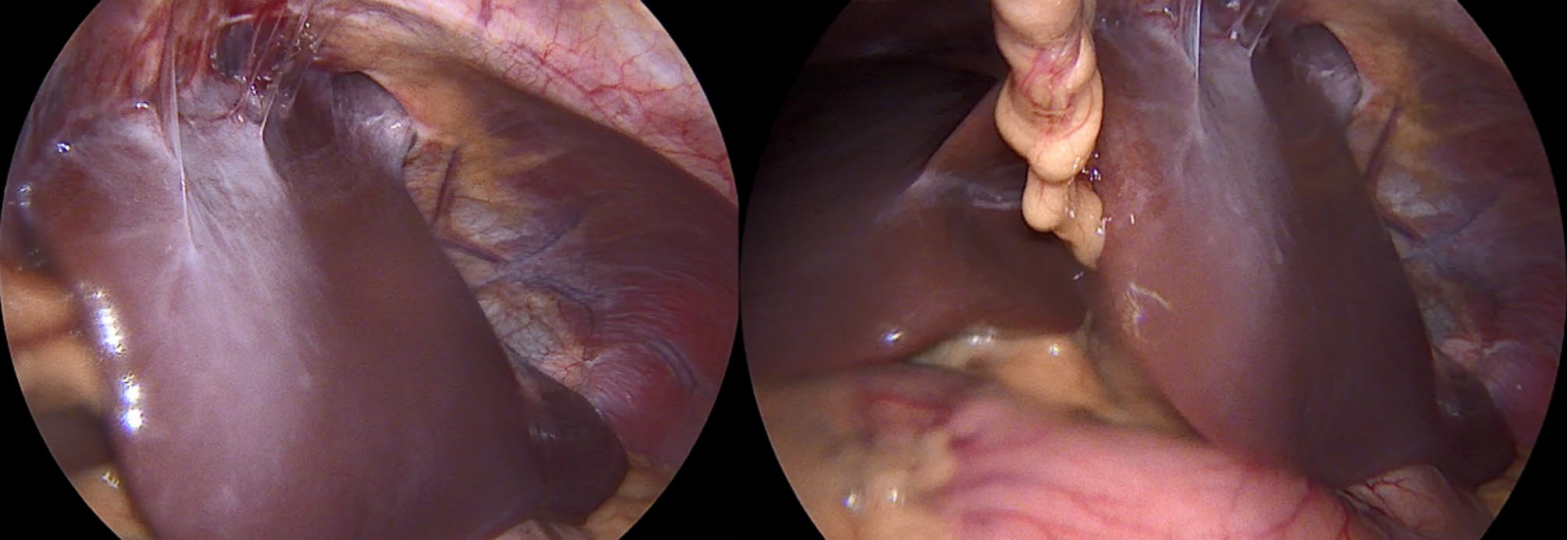
Intraoperative images of initial findings with segment 2 liver protrusion through diaphragmatic hernia from different angles

**Fig. 3:**
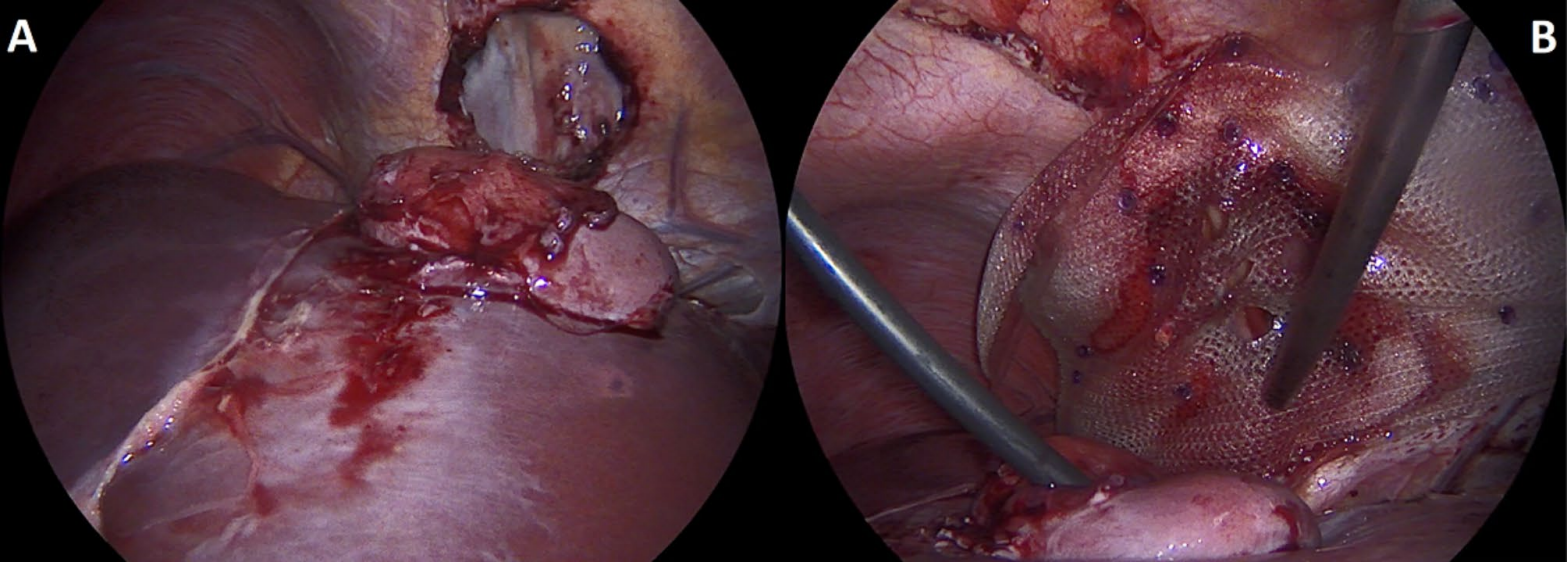
Intraoperative images of surgical reconstruction of intrathoracic liver herniation. (*A: Mobilisation of the liver from the pericardial sac with the visualisation of 9 × 6 cm diaphragmatic defect, B: Placement of the perforated Parietene™ composite mesh*)

A 22-year-old Caucasian male was admitted to the hospital with sings of dyspnoea, shortness of breath on exertion, dry cough, dyspeptic syndrome and seldom night sweats. Patient′s medical history revealed COVID-19 infection 1 year prior, currently with no drug history and findings of enlarged cardiac shadow and mediastinum on the initial chest x-ray. Performed electrocardiography was within standards and echocardiography showed pericardial effusion without the signs of cardiac tamponade at the time. Computed tomography (CT) scans of the chest showed enlarged and multiplied mediastinal lymph nodes, a pathologic mass in the right pulmonary hilum with pericardial effusion. Follow up echocardiography showed progression of the pericardial effusion, therefore the patient underwent pericardial fenestration and the Chamberlain procedure with biopsy. A short incision was made, after preparation in the epigastrium, the pericardium and peritoneum was identified. The pericardial effusion was drained out with the total amount of 700ml of serosangvinous fluid, with subsequent formation of a 2 × 3 cm window between pericardium and peritoneum. The postoperative period was complicated by secondary wound healing. The histology report confirmed the diagnosis of the classical Hodgkin lymphoma. Thereafter, the patient was treated with the BEACOPP (bleomycin, etoposide, doxorubicin hydrochloride (Adriamycin), cyclophosphamide, vincristine (Oncovin), procarbazine and prednisone) regimen of which he underwent six cycles. Two weeks after the last cycle of chemotherapy (approx. 5 months after surgery) the patient displayed the following symptoms for two days; dyspnoea and chest pain with occasional irradiation to the neck. An electrocardiography was performed, which showed only mild tachycardia, while the chest x-ray showed enlargement of the heart. The subsequent echocardiography showed a tumorous mass manifesting in the pericardium, posing as tumour arising from the pericardium or from the liver. The laboratory findings revealed neutrophilic leucocytosis, after administration of granulocyte colony stimulating factor with otherwise unremarkable findings. Heart CT scans showed a retrosternal and subxiphoidal hernia with liver protrusion towards the pericardium. Initially the symptoms were managed conservatively and the patient was discharged home. He underwent scheduled PET/CT (positron emission tomography / computed tomography), which confirmed remission of the Hodgkin lymphoma and findings of the segment 2 liver protrusion into the thorax (Fig. 1). Therefore the patient was scheduled for a planned surgery which was approved by the attending haematologist. When the patient was admitted to our surgical ward a physical examination produced no remarkable findings. The next day, he underwent laparoscopic surgery under general anaesthesia, with findings within the normal range in the abdominal cavity, beside herniation of the liver into the thorax. During the procedure, we performed mobilisation of the liver from the pericardium, which revealed the defect with size of approx. 9 × 6 cm, mobilisation of the peritoneal attachments of the liver. Finally, the perforated Parietene™ composite mesh composed on one side from macroporous polypropylene mesh and the absorbable synthetic film on the other was placed (Figures 2 and 3). The implanted mesh was manually perforated before placement, in order to preserve the original function of pericardial fenestration. At the end of the surgery single Redon drain was placed in the pericardium. The postoperative period was unremarkable. Follow up echocardiography was performed with the findings of minimal residual pericardial effusion complemented with minimal output of the Redon drainage, which was then extracted and the patient was discharged to outpatient care. Two months after surgery the patient is well and without any difficulties, in further observation of his attending haematologist.

## Discussion

The diaphragmatic herniation requires a defect in the diaphragm and can be either congenital or acquired. Congenital diaphragmatic hernia has the prevalence of 2.3 in 10 000 births and is often associated with other congenital anomalies [5, 6]. On the other side the acquired diaphragmatic hernias develop mostly after a traumatic injury. Another group of acquired diaphragmatic hernias is herniation formed after a surgical procedure e.g. gastroesophageal surgery with the incidence of hiatal intraabdominal viscera herniation being about 3% [7].

Liver herniation is quite rare with the first case described in 1908 [8]. The herniated liver often imitates intrathoracic tumour as it is described in multiple reports, which are consistent with the initial findings in our patient [8, 9]. However, utilizing chest and abdominal CT scans should produce sufficient explanation with the diagnosis of the liver herniation into the thoracic cavity. Most reported cases of liver herniation develop after an injury of the diaphragm. However 1% of diaphragmatic ruptures are spontaneous and may be, for example a part of catamenial pneumothorax with subsequent liver herniation [9, 10]. One case study reports a case, of a diaphragmatic rupture with herniation of the liver due to increased transdiaphragmatic pressure, caused by paroxysmal cough due to gastroesophageal reflux disease [9]. Moussa et al. describes liver herniation in a patient with a history of pericardial window. In their case study the patient was admitted to the hospital with intermittent epigastric pain, with acute exacerbation on the third day of hospitalization alongside a subsequent diagnosis of liver volvulus with intrathoracic herniation. The treatment consisted of laparoscopic reduction of the hernial sac containing the left lobe of liver, stomach and transverse colon with subsequent repair of the diaphragm using interrupted sutures and mesh implant, while keeping the original purpose of the pericardial window [10].

The condition of our patient suddenly worsened approximately five months into the treatment of the pericardial effusion and Hodgkin lymphoma. It lasted for few days with the major complaint of dyspnoea and chest pain. The performed imaging studies showed protruding liver parenchyma into the pericardium. Following the confirmation of the success of the chemotherapeutic treatment the patient was scheduled for laparoscopic hernia repair with the hernial sac reduction and diaphragmatic repair with perforated mesh placement, in order to keep the functionality of the original pericardial fenestration.

We believe the aetiology of this complication to be multifactorial. Altogether the patient underwent surgical procedure with superficial secondary wound healing. Furthermore living an active youthful life could add to increased level of intraabdominal to intrathoracic pressure gradient. Confirmed haematologic malignancy with mediastinal lymphadenopathy and pulmonary infiltration causing clinical signs of dyspnoea and cough could potentially add towards increased intraabdominal to intrathoracic pressure ratio as well. The underlying disease could have potentially affected the quality of connective and muscular tissue and therefore unfortunately we cannot precisely determine the original cause of this surgical complication.

## Conclusion

The literature mentions of liver herniation after pericardial fenestration are scarce and according to our knowledge, based on thorough literature search of PubMed and Google Scholar databases, this may be just a second case study describing such a case. Therefore, the aim of this report was to bring attention to such clinical scenario with its clinical presentation, treatment and potential differential diagnosis of intrathoracic masses.

## Data Availability

The authors can confirm that all relevant data are included in the article.

## List of abbreviations

CT: computed tomography
BEACOPP: chemotherapy regimen consisting of: bleomycin, etoposide, doxorubicin hydrochloride (Adriamycin), cyclophosphamide, vincristine (Oncovin), procarbazine and prednisone
PET/CT: positron emission tomography / computed tomography
SCARE: Surgical CAse REport
